# Strong Electron Localization in Tin Halide Perovskites

**DOI:** 10.1021/acs.jpclett.1c01326

**Published:** 2021-06-01

**Authors:** Hassan Ouhbi, Francesco Ambrosio, Filippo De Angelis, Julia Wiktor

**Affiliations:** † Department of Physics, Chalmers University of Technology, SE-412 96 Gothenburg, Sweden; ‡ Computational Laboratory for Hybrid/Organic Photovoltaics (CLHYO), Istituto CNR di Scienze e Tecnologie Chimicie “Giulio Natta” (CNR-SCITEC), Via Elce di Sotto 8, 06123 Perugia, Italy; ¶ CNST@Polimi, Istituto Italiano di Tecnologia, Via Pascoli 70/3, Milano, Italy; § Department of Chemistry, Biology and Biotechnology, University of Perugia, Via Elce di Sotto 8, 06123 Perugia, Italy; ∥ CompuNet, Istituto Italiano di Tecnologia, Via Morego 30, 16163 Genova, Italy

## Abstract

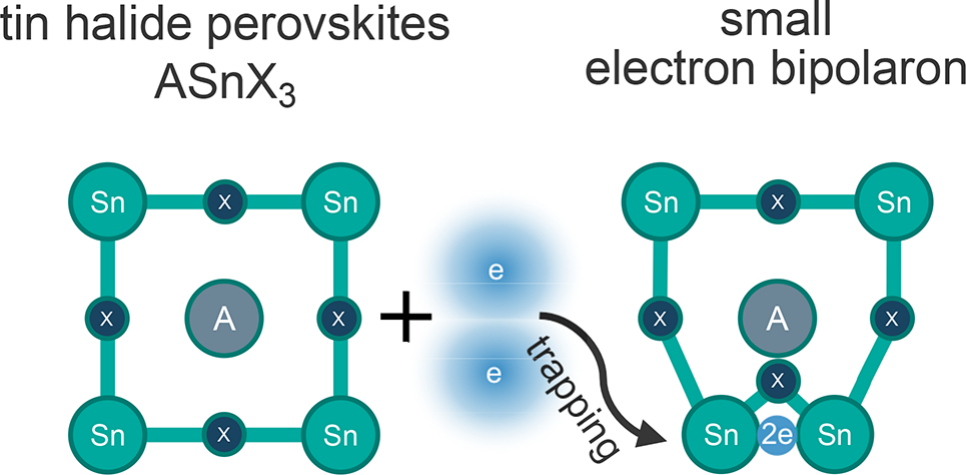

Tin halide perovskites (THPs) have been established as a lower-toxicity alternative to lead halide perovskites. In spite of the increasing interest, the behavior of photoexcited charges has not been well understood in this class of materials. We here investigate the behavior of excess electrons in a series of tin halide perovskites by employing advanced electronic-structure calculations. We first focus on CsSnBr_3_ and show that electron localization is favorable in this compound and that bipolaronic states are the most stable form of self-trapped electrons. We then extend the analysis to CsSnI_3_, CsSnCl_3_, MASnBr_3_, FASnBr_3_, and DMASnBr_3_ and show that electron bipolarons are stable in all these compounds, thus indicating that strong electron localization is recurrent in THPs.

Solar cells based on metal halide perovskites show promising photovoltaic properties with power conversion efficiencies improving rapidly over the past few years and now exceeding 25%.^1^ The highest efficiencies to date have been obtained with compounds containing lead (lead halide perovskites, LHPs), a toxic element.^2−5^ Tin halide perovskites (THPs) exhibit a lower toxicity^6^ and are considered as one of the most promising replacement of LHPs because of their high absorption coefficients, long lifetimes of photogenerated charges, and low impact of defects.^7−10^ The main issue hindering the successful use of THPs is related to their stability, which is hampered by charge-trapping processes. In fact, It has been shown that THPs have stronger tendency to localize extra charges.^11^ This characteristic, in conjunction with the facile oxidation of surface Sn(II) to Sn(IV),^12^ leading to the formation of secondary phases, is at the root of the poor thermodynamic stability of THPs. Such a drawback, intrinsic to tin chemistry, might be circumvented by either deploying surface passivation strategies^10^ or by alloying tin with lead^13^ in lead-alleviated perovskites. However, these strategies address mainly hole-trapping processes while not accounting for electron localization, which may play a major role in THPs, as their band edges are generally closer to the vacuum level with respect to LHPs.^10^ Therefore, a detailed analysis of the electron-trapping phenomena in LHPs is paramount for development of their electronic properties toward the realization of highly efficient devices for photovoltaics.

In the present work, we study the behavior of excess electrons in THPs through advanced ab initio calculations. We first consider single- and double-electron polarons in “polymorphous”^14^ cubic structures of CsSnBr_3_ and then extend the analysis to other THPs. We show that double polarons (bipolarons), associated with the formation of Sn–Sn bonds and leading to strong electron-trapping, are stable in all the studied THPs, thus indicating a common behavior for this class of materials.

We first focus on CsSnBr_3_ as a representative tin-based halide perovskite. This compound adopts a cubic structure at room temperature.^15^ Modeling the cubic structure of halide perovskites is counterintuitively a challenging issue. The effectively cubic structure of a material could suggest that a highly symmetric model can be used to describe the structure. However, because this is a high-temperature phase, the symmetric structure is preserved only on average, while locally strong distortions are present. These instantaneous distortions have been assessed for instance through molecular dynamics^16^ and have been shown to affect the electronic structure of the material significantly. The problem also manifests itself when supercells of the symmetric cubic phase are constructed and relaxed at 0 K. Zhao et al. has shown that in such a supercell a distribution of different low-symmetry local motifs can be observed, called “polymorphous networks”.^14^ This means that a single structure might not be enough to study structural and electronic properties of cubic perovskites.

In the present study we use the cp2k package^17,18^ to generate the various polymorphous structures, both neutral and charged systems. The calculations are performed at the hybrid functional level, using the PBE0(α).^19^ In the PBE0(α) calculations for CsSnBr_3_ we set the α parameter to 0.26, as determined in ref (20) based on the generalized Koopmans’ theorem. We consider a 4 × 4 × 4 cubic supercell containing 320 atoms, with the experimental lattice parameter of *a* = 5.8043 Å.^15^ Additional computational details are given in the Supporting Information.

To overcome the issue of the instability of the perfectly cubic structure at 0 K,^14^ we construct 10 structures with different initial random atomic displacements (up to 0.15 Å). This number of models is enough to achieve good statistics, in accord with ref (14) showing that the electronic structure varies only slightly between the structures. The structures are first fully relaxed before introducing extra charges. To find the configurations of the single-electron polaron, we add one extra charge to each of the 10 neutral structures, beginning the relaxation with PBE0(α = 0.5) to overcome possible energy barriers for charge localization. Once the charge is localized, we continue the relaxation with PBE0(α = 0.26). To form double-electron polarons, in each neutral structure we identify the pair of Sn atoms with the smallest distance. We then reduce the Sn–Sn separation to about 3.2 Å, while displacing the middle Br, based on bipolaronic geometries of CsPbBr_3_ found in ref (21). Finally, we relax the geometry completely with PBE0(α = 0.26).

Because the cp2k package does not allow taking into account the effect of spin–orbit coupling (SOC), which can be significant in tin-based perovskites, we perform additional calculations in the Vienna *ab initio* package (vasp).^22,23^ The calculations are carried out on top of the final geometries, as achieved with the cp2k package. Additional details related to these calculations are given in the Supporting Information. From vasp calculations we extract the difference between formation energies of polarons with and without SOC, at the PBE level. The energy differences are then added to the formation energies found with the cp2k package, following
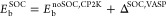
1with
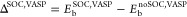
2To verify the validity of including the SOC effect calculated at the PBE level on fixed geometries obtained without SOC in cp2k, we also perform one calculation using the full PBE0+SOC method in vasp, including the geometry optimization. For the bipolaron in CsSnBr_3_, the formation energies obtained with the two approaches differ by only 0.03 eV.

We now focus on single-electron polarons in CsSnBr_3_. In all studied structures, we observe electron localization related to the elongation of three Sn–Br bonds. The polaronic configuration is highly asymmetrical, with the elongated bonds measuring on average 3.37, 3.71, and 4.13 Å, compared to about 2.91 Å in the neutral structures. A representative polaronic configuration is shown in Figure 1a. The formation energies of single-electron polarons are calculated as follows:
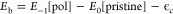
3where *E*_–1_[pol] is the total energy of the relaxed supercell containing the single-electron polaron, *E*_0_[pristine] the energy of pristine CsSnBr_3_, and ϵ_c_ the position of the conduction band minimum (CBM). We here neglect the electrostatic finite-size correction because of the high dielectric constant (68.3^15^) of CsSnBr_3_. We note that in this notation, a negative formation energy indicates that the polaronic state is energetically favorable.

**Figure 1:**
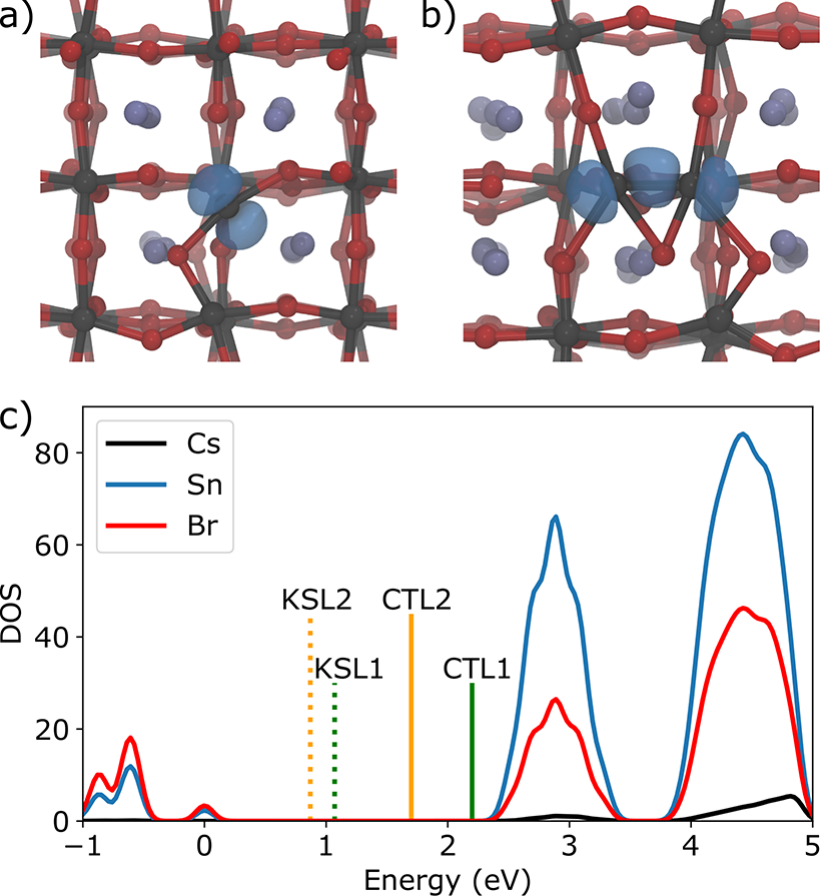
Representative isodensities (in blue, at 20% of the maximum value) of the (a) single- and (b) double-electron polarons in CsSnBr_3_. Cs, Sn, and Br atoms are shown in violet, gray, and red, respectively. (c) Partial densities of states of CsSnBr_3_, aligned with the Kohn–Sham and charge transition levels. KSL1 and CTL1 correspond to the single-electron polarons and KSL2 and CTL2 to the bipolaron.

Without SOC effects, we find the formation energy of the single-electron polaron in CsSnBr_3_ to amount to, on average, −0.30 ± 0.05 eV, suggesting significant stability of the polaronic state. The incertitude is calculated as the mean absolute error (MAE) for the 10 structures. The single-electron polaron is associated with a one-particle Kohn–Sham level (KSL) found at 1.43 eV below the conduction band minimum, as shown in Figure 1b. Upon inclusion of the SOC effect, the formation energy is increased by about 0.24 eV, leading to the final value of −0.06 ± 0.05 eV, suggesting only a weak stability of single-electron polarons in CsSnBr_3_. We note that in some of the polymorphous models the single-electron polarons are particularly stable (with the lowest value of the formation energy of −0.17 eV). This suggests that the charge localization in CsSnBr_3_ can be dynamically stabilized by favorable local distortions, similarly to what was observed in CsPbBr_3_.^21^

Next, we focus on double-electron polarons. In CsPbBr_3_, various bipolaronic states were identified, in which two electrons localized between neighboring metal atoms. We find similar configurations in CsSnBr_3_, one of which is given in Figure 1. In all 10 considered structures, a bond between two Sn atoms of on average 3.07 Å is formed, while the middle Br atom is strongly shifted in the direction perpendicular to the Sn–Sn bond. We note that the bipolaronic configuration can also be considered as a complex of a Br interstitial (I_Br_^–^) and a Br vacancy (V_Br_^–^), both in a negative charge state, because one Br atom leaves its initial position in the perovskite lattice and shifts toward the interstitial site. This corresponds to an excited state of a Frenkel pair, which is formally a complex of V_Br_^+^ and I_Br_^–^.

For the bipolaronic configurations we calculate the formation energy per charge as
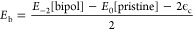
4where *E*_–2_[bipol] is the total energy of the relaxed supercell containing the bipolaron, and the other quantities are defined as in eq 3. We find that without SOC, the total energy of the bipolaronic state is on average 1.59 ± 0.07 eV lower than that of a system with two delocalized electrons. This corresponds to the formation energy of −0.80 ± 0.03 eV per electron, significantly lower than the *E*_b_ of the single polaron (− 0.30 ± 0.05 eV). The alignment between the charge transition levels corresponding to the single- and double-electron polaron (without SOC) is also shown in Figure 1c), where we also plot the partial densities of states of CsSnBr_3_. We find that both the one-particle Kohn–Sham level (KSL) and the charge transition level (CTL) of the bipolaron lies deeper within the band gap. After the inclusion of the SOC effect, the formation energy of the bipolaron is increased by about 0.25 eV, to −0.54 ± 0.03 eV per electron. This large magnitude of the formation energy suggests that the electrons in CsSnBr_3_ are strongly trapped as bipolarons. We note that electron localization is much more favorable in CsSnBr_3_ than in CsPbBr_3_, where the bipolaronic states were found to be unstable at 0 K. .^21^ This is related to the higher position of the conduction band in the lead-based perovskites,^10^ which favors electron trapping. At the same time, the high position of the valence band in tin-based perovskites disfavors hole trapping in the material. We note that we have considered the possibility of hole trapping in CsSnBr_3_ through the formation of both Sn(III) and Sn(IV) ions, Br–Br dimers, or bromine Frenkel defects, but we have found no stable localized states associated with extra holes, in line with previous studies.^10,13^

We also note that the low stability of the single polaronic states may significantly limit the lifetime of the single-polaron state, in turn reducing the probability of capture of a second electron to form the bipolaron. We therefore surmise that electron bipolarons will be mainly formed under high irradiation regimes, i.e. upon establishment of a significant carrier density in the perovskite. As previously found for both lead- and tin-halide perovskites, charge trapping at defects at surfaces and grain boundaries may stabilize singly trapped charges,^12,13,24^ possibly leading to a preferential channel for bipolaron formation.

Because halide perovskites can adapt different structures, we next assess the effect of the phase on polaron stability. This is done by making a comparison with CsSnBr_3_ in the orthorhombic structure. In this case we consider a 2 × 2 × 2 repetition of the unit cell with experimental lattice parameters *a* = 8.1965, *b* = 11.583, and *c* = 8.0243 Å,^15^ containing 160 atoms. For the orthorhombic phase, differently from the cubic structure, only one model is needed. For the single-electron polaron we find the formation energy of −0.25 eV before including SOC effects. Once SOC is included, this value is increased to −0.01 eV. In the case of the bipolaron, without SOC we find the formation energy of −0.60 eV per electron. When relativistic effects are included, the stability of the bipolaron is reduced and the formation energy amounts to −0.36 eV. This is by about 0.18 eV higher than the value found in the cubic phase (−0.54 eV) suggesting that the polaronic states are less stable in the orthorhombic structure of CsSnBr_3_. In both phases we find that the bipolaronic states are much more stable than the single-electron polarons; therefore, we focus only on this type of localization in the rest of our study.

We now extend our analysis to other compounds, and we verify if the strong electron trapping in the bipolaronic state is a more general phenomenon of THPs. We consider CsSnI_3_ and CsSnCl_3_ to assess the effect of the halogen atoms and MASnBr_3_, FASnBr_3_, and DMASnBr_3_ to evaluate that of the cation. In Table 1 we summarize the structural properties of the considered compounds and the α parameters used in the PBE0 functional. The values are taken from previous studies either based on the generalized Koopmans theorem^20^ or chosen to reproduce the experimental band gap of the material (reference values given in Table 1). We note that in the cases of CsSnCl_3_, MASnBr_3_, and FASnBr_3_ which are cubic, we take only one polymorphous model, because in CsSnBr_3_ we observed a small variation of the bipolaron formation energy between the considered models. The formation energies of the bipolarons are given in Table 2, in which we report both the values neglecting and including SOC. The trend within the set of compounds containing Cs can be directly correlated with the size of the band gap, with the smallest bipolaron stability found in CsSnI_3_ (experimental band gap of 1.3 eV^25^) and the largest in CsSnCl_3_ (experimental band gap of 2.8 eV^26^). The trend can also be observed from the alignment of polaronic levels with the densities of states of the various perovskites shown in Figure S1. The change of cation has a less straightforward effect on the bipolaron formation energy, in line with what was observed in lead halide perovskites.^27^ For instance, even though the band gap of DMASnBr_3_ is significantly larger than that of CsSnBr_3_ (2.9 eV^28^ compared to 1.8 eV^29^), the bipolaron is less stable in the former material. This can be explained based on a larger volume per unitary formula, increasing the deformation cost of bringing two Sn atoms closer, thus suggesting another handle to tune the electronic properties of THPs. Nevertheless, we observe that the bipolarons are stable in all considered tin-based halide perovskites. As a consequence, we conclude that in general the excess electrons in THPs can be trapped and therefore their mobilities limited, which could be detrimental to the performance of optoelectronic devices. On the other hand, charge localization could be beneficial, because polaron formation extends charge carrier lifetimes by reducing the overlap between hole and electron wave functions.^30^ The polaron formation has been shown to reduce both monomolecular^31^ and bimolecular^32^ recombination rates in lead halide perovskites. However, the aforementioned observations have been made for more delocalized polarons in MAPbI_3_, and the effect of small polaron formation in THPs on the charge carrier lifetimes should be further examined.

**Table 1:** Space Groups and Lattice Parameters of the Considered Tin-Based Halide Perovskites^a^

	space group	*a* (Å)	*b* (Å)	*c* (Å)	α	*E*_gap_^exp.^ (eV)
CsSnI_3_	*Pnma*^b^	8.69	12.38	8.64	0.23	1.3^b^
CsSnBr_3_	*Pm*3̅*m*^c^	5.80	5.80	5.80	0.26	1.8^c^
CsSnCl_3_	*Pm*3̅*m*^d^	5.56	5.56	5.56	0.35	2.8^d^
MASnBr_3_	*Pm*3̅*m*^e^	5.91	5.91	5.91	0.20	2.0^e^
FASnBr_3_	*Pm*3̅*m*^f^	6.00	6.00	6.00	0.23	2.4^f^
DMASnBr_3_	*Pbca*^g^	6.15	6.08	6.08	0.18	2.9^g^

a α is the fraction of Fock exchange incorporated in the PBE0(α) functional.

b Experimental data come from ref (7).

c Experimental data come from refs (15 and 29).

d Experimental data come from refs (33 and 26).

e Experimental data come from ref (34).

f Experimental data come from ref (35).

g Experimental data come from ref (36).

**Table 2:** Formation Energies (per Charge) of the Electron Bipolarons in Various Tin-Based Halide Perovskites^a^

	*E*_b_ (eV) without SOC	*E*_b_ (eV) with SOC	*d*_Sn–Sn_ (Å)
CsSnI_3_	–0.35	–0.12	3.12
CsSnBr_3_	–0.80	–0.54	3.07
CsSnCl_3_	–1.43	–1.23	3.03
CsSnBr_3_	–0.80	–0.54	3.07
MASnBr_3_	–0.46	–0.32	3.13
FASnBr_3_	– 0.68	– 0.46	3.09
DMASnBr_3_	–0.51	–0.36	3.15

a Values before and after including SOC are given. The Sn–Sn bond length (*d*_Sn–Sn_ (Å)) is also given.

In conclusion, we studied the behavior of excess electrons in tin halide perovskites using hybrid density functional theory. We first focused on cubic CsSnBr_3_, in which we studied both single- and double-electron polarons. We considered a set of “polymorphous” cubic models to overcome the problems of instability of the cubic perovskite phase at 0 K. We observed that while spin–orbit coupling significantly reduces the stability of polarons in CsSnBr_3_, they still represent the favorable form of excess electrons. Having found that the bipolaronic states are more stable than two isolated single-electron polarons, we extended our study to other THPs, namely to CsSnI_3_, CsSnCl_3_, MASnBr_3_, FASnBr_3_, and DMASnBr_3_ (Figure 2), in order to assess the effect of both the halogen and the A-site cation. We observed that bipolaronic states are energetically favorable in all these compounds and can lead to strong electron trapping and reduced mobility of charge carriers. Halogen substitution has been found to induce a straightforward effect on bipolaron stabilization, which was found to increase from I to Cl, following the higher band gap of the material. In contrast, the effect of the A-site cation is less obvious. Because of the larger variation in the volume associated with the change in the A-site cation, the gap–polaron correlation is not preserved in this case. In fact, less compact THPs may entail a larger deformation cost for the formation of the Sn–Sn, thus lowering the stability of the bipolaron. Overall, our results demonstrate that strong electron trapping is recurrent in THPs and may limit their application in optoelectronic devices.

**Figure 2:**
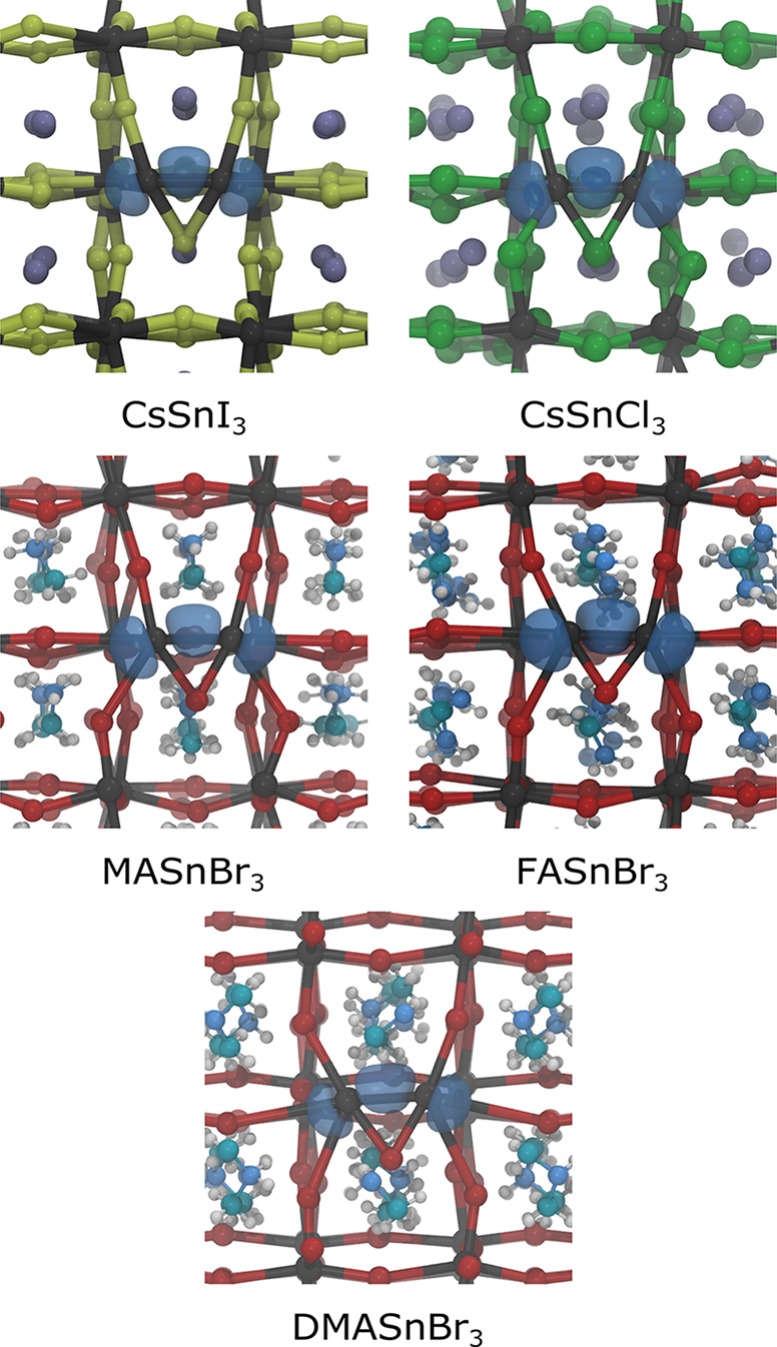
Isodensities (in blue, at 20% of the maximum value) of the bipolaronic states in CsSnI_3_, CsSnCl_3_, MASnBr_3_, FASnBr_3_, and DMASnBr_3_.

## Supporting Information Available

Computational details, assessment of finite-size corrections, justification of the parametrization of the hybrid density functionals, and densities of states of all considered tin halide perovskites with the Kohn–Sham and charge transition levels corresponding to the bipolaronic states (PDF)
