# Supplementary material for: Strong Electron Localization in Tin Halide Perovskites
Source: J Phys Chem Lett. 2021 Jun 1;12(22):5339–43. doi: 10.1021/acs.jpclett.1c01326 (PMC8280731; doi:10.1021/acs.jpclett.1c01326)
Supplement: Supplementary file 1 — jz1c01326_si_001.pdf [file jz1c01326_si_001.pdf]

Supporting Information: Strong Electron Localization in Tin Halide Perovskites

Hassan Ouhbi,[†] Francesco Ambrosio,[‡,¶] Filippo De Angelis,[§,‡,‖] and Julia Wiktor[*,†]

†*Department of Physics, Chalmers University of Technology, SE-412 96 Gothenburg, Sweden*

‡*Computational Laboratory for Hybrid/Organic Photovoltaics (CLHYO), Istituto CNR di Scienze e Tecnologie Chimicie "Giulio Natta" (CNR-SCITEC), Via Elce di Sotto 8, 06123, Perugia, Italy*

¶*CNST@Polimi, Istituto Italiano di Tecnologia, Via Pascoli 70/3, Milano, Italy*

§*Department of Chemistry, Biology and Biotechnology, University of Perugia, Via Elce di Sotto 8, 06123, Perugia, Italy*

‖*CompuNet, Istituto Italiano di Tecnologia, Via Morego 30, 16163 Genova, Italy*

E-mail: julia.wiktor@chalmers.se

Methods

Computational details

CP2K

Within the CP2K package, we employ atom-centered Gaussian-type basis functions to describe the electronic orbitals and an auxiliary plane-wave basis set to re-expand the electron density. We use DZVP-MOLOPT basis sets[S1] and a cutoff energy of 200 Ha. Core-valence interactions are described by Goedecker-Teter-Hutter pseudopotentials.[S2] We sample the Brillouin zone at the sole Γ point. Two ways of accelerating hybrid functional calculations are applied within CP2K. First, we use the PBE0 functional with truncated Coulomb interaction and including long-range corrections. Second, we apply the auxiliary density matrix method (ADMM),[S3] with the cFIT auxiliary basis set, to speed up the calculation of exact exchange in hybrid functional calculations. During geometry optimization, we relax atomic positions of both neutral models and polaronic structures until the magnitude of forces on atoms become less than about 0.02 eV/Å.

VASP

Spin-orbit coupling (SOC) has been shown to significantly affect the energetics of polaronic states in halide perovskites.[S4] Therefore, it is necessary to account for this effect when determining stability of localized states in the materials considered here. Since calculations including SOC are not possible within the CP2K package, we perform additional calculations within VASP including relativistic corrections. Therein, we employ the projector augmented wave method.[S5] We use PAW data sets with the following valence orbitals: H [1s], C [2s2p], N [2s2p], Cs [5s5p6s], Sn [5s5p], Cl [3s3p], Br [4s4p], and I [5s5p]. We set the plane-wave cut-off to 300 eV and sample the Brillouin zone at the Γ point. Within VASP we perform four types of calculations, with and without SOC for the pristine structures, and with and without SOC for polaronic structures. By extracting differences in total energies between

relevant calculations, we find the corrections to the formation energies of the polaronic states due to SOC, which are then applied on top of the results from CP2K. The corrections due to SOC are calculated at the PBE level of theory. This is justified by the fact that spin-orbit splittings of the electronic levels do not significantly change with the level of theory.[S6]

Choice of the α parameter in the hybrid density functionals

Calculated stability of polaronic states strongly depends on the parametrization of the hybrid functional.[S4] To correctly describe the localized electronic states, a functional exactly correcting the self-interaction correction needs to be applied.[S7] A direct way of correcting self-interaction errors is to make sure that the functional satisfies Koopmans' condition,[S8] which states that the single-particle level of a localized state should not depend on its occupation. In the present study, we use the α parameters previously determined by applying the Koopmans' condition to halogen vacancies in $CsSnCl_3$, $CsSnBr_3$, and $CsSnI_3$.[S9] Since α parameters achieved in this way have not been reported for $MASnBr_3$, $FASnBr_3$, and $DMASnBr_3$, we choose an indirect approach in these cases. It has been shown that in the wide range of halide perovskites the fhybrid functionals that satisfy Koopmans' condition, also reproduce well the experimental band gaps when appropriate effects are accounted for.[S9] Therefore, for these materials we set the α parameter to a value that matches the experimental band gap increased by spin-orbit coupling effects.

Finite-size corrections

In the present study, we use the approach based on periodically repeated charged supercells to study electron polarons. In such calculations, a homogeneous neutralizing background charge (jellium) is included to avoid the divergence of the Coulomb interaction. Interactions between periodic images of the extra charge, as well as between the polarons and the neutralizing background can lead to errors in total energies.[S10,S11] We evaluated the finite-size corrections in the case of the single electron polaron in $CsSnBr_3$, considering the static dielectric constant

$\epsilon_0 = 68.3$.[S12] We found that, due to the high screening in the material and the large size of the used supercell (320 atoms), the correction amounts to 0.01 eV only. We therefore neglect the finite-size corrections in the present study.

Densities of states and polaronic levels

In Figure S1 we show densities of states of the considered tin halide perovskites. The VBM was set to zero for all materials. We include both the Kohn-Sham (one particle) and the charge transition levels corresponding to the bipolaronic states. The DOS are calculated without SOC, within PBE0(α), with values of α as given in Table 1 in the main text.

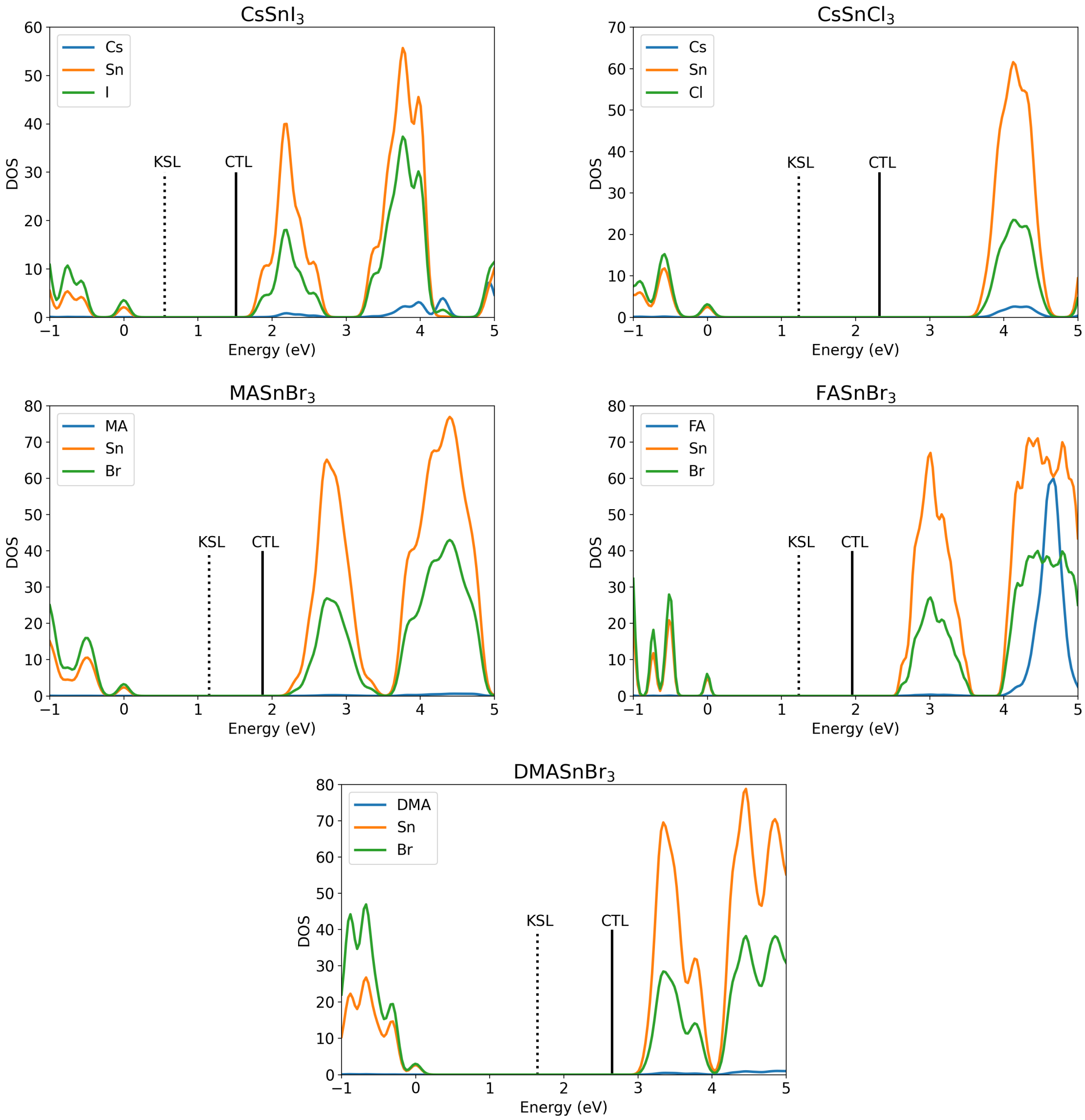

Figure S1: Densities of states of the considered tin halide perovskites. Dotted lines mark the Kohn-Sham levels (KSL) related to the bipolaronic states. Solid lines mark the charge transition levels (CTL) related to the bipolarons.

References

(S1) VandeVondele, J.; Hutter, J. Gaussian Basis Sets for Accurate Calculations on Molecular Systems in Gas and Condensed Phases. *J. Chem. Phys.* **2007**, *127*, 114105.

(S2) Goedecker, S.; Teter, M.; Hutter, J. Separable Dual-Space Gaussian Pseudopotentials. *Phys. Rev. B* **1996**, *54*, 1703.

(S3) Guidon, M.; Hutter, J.; VandeVondele, J. Auxiliary Density Matrix Methods for Hartree- Fock Exchange Calculations. *J. Chem. Theory Comput.* **2010**, *6*, 2348–2364.

(S4) Österbacka, N.; Erhart, P.; Falletta, S.; Pasquarello, A.; Wiktor, J. Small Electron Polarons in $CsPbBr_3$: Competition Between Electron Localization and Delocalization. *Chem. Mater.* **2020**, *32*, 8393–8400.

(S5) Blöchl, P. E. Projector Augmented-Wave Method. *Phys. Rev. B* **1994**, *50*, 17953.

(S6) Kim, Y.-S.; Hummer, K.; Kresse, G. Accurate Band Structures and Effective Masses for InP, InAs, and InSb Using Hybrid Functionals. *Phys. Rev. B* **2009**, *80*, 035203.

(S7) Zhang, Y.; Yang, W. A Challenge for Density Functionals: Self-Interaction Error Increases for Systems with a Noninteger Number of Electrons. *J. Chem. Phys.* **1998**, *109*, 2604–2608.

(S8) Perdew, J. P.; Parr, R. G.; Levy, M.; Balduz Jr, J. L. Density-functional Theory for Fractional Particle Number: Derivative Discontinuities of the Energy. *Phys. Rev. Lett.* **1982**, *49*, 1691.

(S9) Bischoff, T.; Wiktor, J.; Chen, W.; Pasquarello, A. Nonempirical Hybrid Functionals for Band Gaps of Inorganic Metal-Halide Perovskites. *Phys. Rev. Mater.* **2019**, *3*, 123802.

(S10) Komsa, H.-P.; Rantala, T. T.; Pasquarello, A. Finite-Size Supercell Correction Schemes for Charged Defect Calculations. *Phys. Rev. B* **2012**, *86*, 045112.

(S11) Freysoldt, C.; Grabowski, B.; Hickel, T.; Neugebauer, J.; Kresse, G.; Janotti, A.; Van de Walle, C. G. First-Principles Calculations for Point Defects in Solids. *Rev. Mod. Phys.* **2014**, *86*, 253–305.

(S12) Fabini, D. H.; Laurita, G.; Bechtel, J. S.; Stoumpos, C. C.; Evans, H. A.; Kontos, A. G.; Raptis, Y. S.; Falaras, P.; Van der Ven, A.; Kanatzidis, M. G., et al. Dynamic Stereochemical Activity of the Sn^{2+} Lone Pair in Perovskite $CsSnBr_3$. *J. Am. Chem. Soc.* **2016**, *138*, 11820–11832.
